# The association of spatial T wave axis deviation with incident coronary events. The ARIC cohort

**DOI:** 10.1186/1471-2261-5-2

**Published:** 2005-01-11

**Authors:** Georgeta D Vaidean, Pentti M Rautaharju, Ronald J Prineas, Eric A Whitsel, Lloyd E Chambless, Aaron R Folsom, Wayne D Rosamond, Zhu-Ming Zhang, Richard S Crow, Gerardo Heiss

**Affiliations:** 1 Department of Epidemiology, University of North Carolina at Chapel Hill, USA; 2 Department of Public Health Sciences, Wake Forest University School of Medicine, Winston-Salem, North Carolina, USA; 3 Department of Epidemiology and Department of Medicine, University of North Carolina at Chapel Hill, USA; 4 Department of Biostatistics, University of North Carolina at Chapel Hill, USA; 5 Division of Epidemiology, University of Minnesota, Minneapolis, Minnesota, USA

## Abstract

**Background:**

Although current evidence suggests that the spatial T wave axis captures important information about ventricular repolarization abnormalities, there are only a few and discordant epidemiologic studies addressing the ability of the spatial T wave axis to predict coronary heart disease (CHD) occurrence.

**Methods:**

This prospective study analyzed data from 12,256 middle-aged African American and white men and women, from the Atherosclerosis Risk in Communities Study (ARIC). Following a standardized protocol, resting standard 12-lead, 10-second electrocardiograms were digitized and analyzed with the Marquette GE program. The median follow-up time was 12.1 years; incident coronary heart disease comprised fatal and non-fatal CHD events.

**Results:**

The incidence rate of CHD was 4.26, 4.18, 4.28 and 5.62 per 1000 person-years respectively, across the spatial T wave axis quartiles. Among women for every 10 degrees increase in the spatial T wave axis deviation, there was an estimated increase in the risk of CHD of 1.16 (95% CI 1.04–1.28). After adjustment for age, height, weight, smoking, hypertension, diabetes, QRS axis and minor T wave abnormalities, this hazard rate ratio for women fell to 1.03 (0.92–1.14). The corresponding crude and adjusted hazard ratios for men were 1.05 (95% CI 0.96–1.15) and 0.95 (0.86–1.04) respectively.

**Conclusions:**

In conclusion, this prospective, population-based, bi-ethnic study of men and women free of coronary heart disease at baseline shows that spatial T wave axis deviation is not associated with incident coronary events during long-term follow up. It is doubtful that spatial T wave axis deviation would add benefit in the prediction of CHD events above and beyond the current traditional risk factors.

## Background

Ventricular repolarization abnormalities play an important role in the determination of arrhythmia and sudden cardiac death [1,2] and may reflect subclinical myocardial ischemia changes. The process of repolarization at rest is routinely quantified from standard 12-lead electrocardiogram (ECG), either as time-domain indexes such as the QT interval and its derivations, or as abnormalities of the ST segment or of the T wave. While these indexes have all been reported to be associated to some degree with incident coronary heart disease events, they have some limitations. The widely used QT interval reflects only the temporal aspect of the repolarization, and as currently defined and measured from the 12-lead ECG, QT dispersion has major conceptual and technical limitations [3,4].

Theoretical and experimental studies suggest that ventricular repolarization occurs in a nonlinear and inhomogeneous fashion [5-10]. As a consequence, spatial measures of repolarization that take into account T-wave complexity using the T-wave vector (axis) should be more accurate and useful surface ECG markers of repolarization abnormalities than simple scalar intervals from the ECG, such as the QT interval or QT dispersion [11-16].

Clinical studies have shown that the T wave axis reflects changes associated with autonomic adaptive or maladaptive influences [17], systemic hypertension [18,19], coronary occlusion [20], and microalbuminuria in individuals without diabetes mellitus [21]. Moreover, it has recently been shown that spatial T wave axis deviation has good measurement properties and is repeatable [22], a finding that supports its use in clinical and epidemiological research.

Two prospective population-based reports on T wave axis deviation measured from standard 12-lead ECGs in older populations (the Rotterdam study [23], and Cardiovascular Health Study (CHS) [24] suggest that it is an indicator of increased risk of coronary heart disease and total mortality, independent of other cardiovascular risk factors. In the cohort of high-risk, middle-aged men from the Multiple Risk Factor Intervention Trial (MRFIT), baseline spatial T wave axis deviation was not significantly associated with incident coronary events, although the change over time in the spatial T wave axis deviation was reported to be associated with incident events on long-term follow-up [25]. These studies suggest that spatial T wave axis deviations capture changes in the ventricular repolarization process that are of potential clinical and epidemiologic importance.

The aim of this study was to assess whether a single base-line measurement of spatial T wave axis deviation is associated with prospectively ascertained coronary events in a population-based, bi-ethnic cohort of middle-aged men and women, to determine if such an association is independent of other risk factors, and to compare its association with that of other indexes of altered repolarization, such as a prolonged QT interval and minor T wave abnormalities.

## Methods

Between 1987 and 1989, the Atherosclerosis Risk in Communities (ARIC) study examined population-based samples of residents aged 45 to 64 years from 4 communities in North Carolina, Mississippi, Minnesota, and Maryland. From the initial sample of ARIC baseline participants (n = 15,792) we excluded those not having an ECG (n = 205), and because of small numbers, self-identified ethnicity other than white or African American (n = 48). Further, we excluded participants likely to present secondary repolarization abnormalities such as myocardial infarction (self-reported or ECG-diagnosed, n = 790), use of digitalis or anti-arrhytmic drugs (n = 115), or pathologies likely to alter the measurement or interpretation of the spatial T wave axis as identified by the Minnesota code (MC), such as major Q or QS waves (MC 1.1, 1.2), ST depression or elevation (MC 4.1 to 4.4 and MC 9.2), negative T waves (MC 5.1 or 5.2), WPW pattern (MC 6.6), ventricular conduction defects (MC 7.1, 7.2, 7.4, and atrial fibrillation or flutter (MC 8.3), (n = 2093). Participants who had ECG evidence or history of myocardial infarction, coronary bypass, or angioplasty) (n = 162) at baseline were excluded from analysis. Participants whose angina status was positive or unknown by the Rose questionnaire were not excluded because the questionnaire's validity, especially in women, has been questioned [26]. The final study sample was 12,256 participants.

### Baseline measurements

#### ECG measurements, processing, and definition of the spatial T wave axis

During a fifteen-minute supine rest, trained and certified technicians positioned disposable Ag/AgCl electrodes and recorded a supine, resting 12-lead, 10-second electrocardiogram using a MAC Personal Cardiographer™ (Marquette Electronics, Inc., Jupiter, FL). Detailed procedures used for electrode placement and skin preparations are described in the Operation Manual [27]. ECGs were digitized at 250 Hz and sent daily via modem to the Epidemiological Cardiology Research (EPICARE) Center, North Carolina. The EPICARE Center, blinded to participant identity processed the ECGs using the most recent version of the Marquette GE program, version 12SL.

Quality assurance procedures were designed specifically for ECG acquisition and processing [27]. These programs included: standardized electrode positions were assured by marking of the skin using a standardized flexible ruler according to a detailed procedure manual. Electrical noise, overall and beat-to-beat electrocardiogram drift were monitored and quality-scored using a five level grading system to assist technicians in identifying unacceptable electrocardiograms and maintaining quality. Technical performance criteria were established and monitored by the EPICARE Center. All technicians were specifically trained and certified. All ECGs were read according to the Minnesota code [28] without knowledge of clinical or demographic data. All resting 12 lead ECGs coded as having key abnormalities by Minnesota codes including any 1-code, any 1-2 code or 2-2, 5-1 or 2 or any 9-2, 6-4, 7-1-1 or 7-2-1 codes and a 10% random sample of all other ECGs read by computer also were visually coded at the Minnesota ECG Reading Center. Adjudication of discrepancies was performed at the Minnesota Center. The computer-assigned codes are used as study data except where adjudication resulted in a code different from the original computer codes.

Spatial T-wave axis was calculated as previously described [24] from integrated T-wave amplitudes of the XYZ leads. Briefly, the inverse transformation by Dower et al. [29] was used to derive Frank's XYZ leads, with the polarity of the Z lead inverted in order to generate QRS and T wave patterns with a more familiar waveform in that lead. Thus, the positive direction of the Z axis is in the anterior direction. Spatial T-wave axis was calculated from the scalar product between the T vector and a unit vector in a normal reference direction (x = 1/√3, y = 1/√3, and z = -1/√3, where x, y and z are the unit vector components in the X, Y and Z directions). T axis expresses spatial T vector deviation from the approximate normal direction of the T vector 45° anteriorly in the XZ plane and at 45° elevation from the Y axis.

The repeatability of the spatial T wave axis deviation was determined using identical study procedures in a group of apparently healthy volunteers with demographic characteristics similar to the ARIC participants. The repeatability of the spatial T wave axis deviation was high with an intra-class correlation coefficient of 0.87 [22]. The Cornell voltage index was determined as the sum of R wave amplitude in leads aVL and the S wave amplitude in lead V3 [30]. The QT interval from the digital 12-lead ECG was determined by the NOVACODE program [31]. An overall QT interval was calculated from the common QRS onset and T offset for all 12 leads together. To attempt correction for the heart rate dependence, we used several approaches: Bazett's and Fridericia's formulae as well as the QT Index. For comparability with other studies we report the QT interval "corrected "by the Bazett's formula [32], in spite of its well-known limitations.

### Covariate measurements

The baseline examination was carried out in 1987–1989 and consisted of a home interview of all potential cohort members including items on cardiovascular risk factors, socioeconomic factors and family medical history. After obtaining informed consent, the clinic examination consisted of medical history interview, blood pressure and anthropometric measurements, venipuncture for blood samples and a 12-lead standard electrocardiogram. Anthropometrics were measured with participants wearing scrub suits and no shoes. Height, measured to the nearest centimeter and weight, measured to the nearest pound, were used to calculate body mass index (BMI). Waist-to-hip ratio was defined as the waist girth at the umbilicus (centimeters) divided by the maximum girth of the hips (centimeters). Smoking status was defined as "current smoker" if the person answered "yes" to both of the following questions: "Have you ever smoked cigarettes? and " Do you now smoke cigarettes?". Cholesterol and triglycerides were measured enzymatically. LDL-cholesterol was calculated. Hypertension was defined as having systolic blood pressure values equal or higher than 140 mmHg, or diastolic blood pressure values equal or higher than 90 mmHg or use of blood pressure lowering medication use in the past two weeks [27].

### Ascertainment and classification of incident CHD cases

Deaths and hospitalization events were ascertained by annual follow-up calls to the cohort members, review of vital records, and community surveillance of hospitalized and fatal events. CHD death was defined as lacking a probable non-CHD cause, and occurring in the context of a recent myocardial infarction, chest pain within 72 hours of death, or a history of CHD. Events were classified independently by two members of the Mortality and Morbidity Classification Committee and discrepancies were adjudicated by a third member. Descriptions of event ascertainment and classification have been published [33,34]. For the present study, we included CHD events occurring between the ARIC baseline examination and December 31, 2000. The median follow-up time was 12.1 years (maximum of 14.1). We defined CHD incidence as (1) a definite or probable MI, (2) a silent MI between examinations ascertained by ECG, or (3) a definite CHD death.

### Data analysis

We treated spatial T wave axis deviation as a continuous variable. For the purpose of comparison, however we categorized the spatial T wave axis deviation using thresholds employed in previously published reports: normal (<30 degrees), borderline (30 to 45 degrees), and abnormal deviation (>=45 degrees). Cox proportional hazards regression [35] was used to model time-to-event in the presence of censoring. Time-at-risk (time-to-event or time-to-censoring) was calculated from the date of the baseline examination to the earliest of the following: date of coronary event, date of death, date of last follow-up contact, or December 31, 2000. Hazard ratios with 95%confidence intervals were derived for each variable. When treating spatial T wave axis as a categorical variable, dummy variables were created. Interactions between spatial T wave axis and gender, QRS axis, minor T wave abnormalities and QT interval, were tested as cross-product terms in the multivariable model. Significance of interaction was assessed based on the likelihood ratio test (p < 0.10). Statistical analyses were conducted for each gender to allow for well-known differences in ventricular repolarization by gender, and because of a statistically significant interaction between spatial T axis and gender on CHD events. SAS version 8.1 (SAS Institute, Cary, North Carolina) was used in all analyses.

## Results

### Sample characteristics

The study population consisted of 12,256 participants, of which 7,143 (58.3%) were women and 3,050 (24.9%) were African American. The mean (SD) age of the study population at baseline was 53.8 (5.7) years. The cohort was followed up for a maximum of 14.1 years [mean (SD) 11.6 (2.3) years]. During this time 653 incident coronary heart disease events occurred. Of the total number of events, 250 coronary heart disease events occurred among women (cumulative incidence 3.5%) and 403 (cumulative incidence 7.9%) among men. The average annual incidence rate was 4.6 per 1000 person-years (95% CI, 4.2–4.9 per 1000 person-years).

### Correlates of spatial T wave axis deviation

The distribution of the spatial T wave axis deviation at baseline was similar for men and women, with a mean (SD) of 23.9 (10.7) degrees in men and 23.2 (11.6) degrees in women. Several traditional cardiovascular risk factors showed graded associations across the spatial T wave axis deviation quartiles, with similar patterns among both men and women (Table 1). Participants of African American ethnicity, patients with hypertension and diabetes, and those with higher Cornell voltage presented larger values of spatial T wave axis deviation; lower values for the spatial T wave axis were observed among participants with larger values of the QRS axis, T wave axis in the frontal plane and among smokers. We considered the frontal plane T wave axis for ensuring comparability with other published studies. Other covariates analyzed were anthropometric measures (weight, BMI, waist-to-hip ratio), systolic and diastolic blood pressure, glucose levels, total-, and LDL-cholesterol, QT interval, the prevalence of minor T wave abnormalities. All showed statistically significant, positive associations with spatial T wave axis quartile. Age was not associated with spatial T wave axis deviation.

**Table 1:** Characteristics of the study population at baseline examination by spatial T wave axis extreme quartiles. The ARIC Study. Mean (SD) and percentages

Variable (units)	**Spatial T wave axis deviation quartile**			
	
	**Women**, n = 7143		**Men**, n = 5133	
	
	T axis quartile 1: 0.33–14.75°	T axis quartile 4: 30.20–60.76°	T axis quartile 1: 0.19–16.19°	T axis quartile 4: 30.41–60.88°
Age (years)	53.5 (5.7)	53.7 (5.6)	54.4 (5.7)	54.4 (5.7)
African-American %	18.4	41.7	13.0	32.1
Height (cm)	162.3 (5.9)	161.9 (6.0)	176.5 (6.2)	175.5 (6.7)
Weight (Lb)	151.7 (32.5)	168.6 (37.8)	182.3 (30.8)	190.6 (31.8)
BMI (kg/m*m)	26.2 (5.4)	29.2 (6.3)	26.6 (4.1)	28.1 (4.3)
WHR	0.88 (0.08)	0.90 (0.08)	0.96 (0.06)	0.96 (0.05)
Smokers %	26	21	32	29
SBP (mmHg)	116.8 (18.2)	122.9 (19.4)	118.8 (15.5)	126.1 (18.5)
DBP (mmHg)	70.3 (10.3)	74.2 (11.1)	73.3 (9.8)	78.2 (11.6)
HTN %	25	43	22	44
Glucose (mmol/L)	5.8 (2.3)	6.1 (2.5)	5.8 (1.4)	6.2 (2.3)
Diabetes %	8	13	7	14
HDL-C (mmol/L)	1.5 (0.4)	1.5 (0.4)	1.2 (0.4)	1.2 (0.4)
LDL-C (mmol/L)	3.5 (1.0)	3.6 (1.0)	3.6 (1.0)	3.6 (1.0)
Total Cholesterol (mmol/L)	5.6 (1.1)	5.7 (1.1)	5.4 (1.0)	5.5 (1.0)
Tri-glyc. (mmol/L)	1.3 (0.8)	1.4 (1.0)	1.6 (1.2)	1.7 (1.1)
QRS axis (degrees)	52.5 (30.0)	32.8 (32.0)	52.1 (34.3)	24.0 (35.9)
Cornell voltage (uV)	926.1 (400.6)	1188.9 (437.4)	1165.6 (44.7)	1489.8 (490.6)
QT interval (ms)	394.2 (27.4)	403.9 (28.3)	395.0 (29.2)	400.1 (29.8)
Heart rate (bpm)	70.2 (9.8)	67.7 (9.9)	62.3 (10.3)	66.7 (10.1)
QTc (ms)	399.2 (18.5)	403.9 (19.1)	415.0 (16.4)	418.5 (18.7)
T axis frontal plane (degrees)	61.5 (12.3)	38.3 (25.4)	62.2 (12.5)	31.4 (26.8)
Minor T wave abn.%	5.3	16.4	3.8	16.8

BMI = Body Mass Index, WHR = waist-to-hip-ratio, QTc = QT interval corrected for heart rate. bpm = beats per minute. SBP, DBP systolic and diastolic blood pressure, HTN, hypertension

Of particular relevance is the association of the spatial T wave axis with hypertension and with the heart rate. The relationship between the spatial T wave axis and hypertension status (according to the JNC-VII classification) is presented in Table 2 by gender and adjusted for age, height and weight. The more pronounced the hypertension status the larger the mean values for the spatial T wave axis. Among the 3860 individuals defined as hypertensive, the mean (SD) values for the spatial T wave axis adjusted for age, height and weight were higher among those with uncontrolled HTN compared to those having the blood pressure levels below the treatment goal: 26.73 (0.27) degrees vs. 25.90 (0.27) (p = 0.03).

**Table 2:** Mean spatial T wave axis deviation adjusted for age, height and weight, by JNC VII classification of blood pressure and gender. The ARIC study

**JNC VII BP stage**	**Women**		**Men**	
	
	N (%)	Mean T axis (SE)	N (%)	Mean T axis (SE)
Normal	3765 (52.73)	9.86 (0.10)	2373 (46.44)	11.23 (0.11)
Pre-HTN	2286 (32.02)	18.29 (0.10)	1911 (37.40)	19.80 (0.11)
Stage 1 HTN	841 (11.78)	25.81 (0.10)	648 (12.68)	26.63 (0.11)
Stage 2 HTN	248 (3.47)	39.04 (0.10)	178 (3.48)	38.13 (0.1)

We found a small but significant inverse association between spatial T wave axis deviation and heart rate. From the first to the fourth T wave axis quartile the mean (SE) values for the heart rate were: 70.24 (0.30), 69.05 (0.23), 69.20 (0.23), 67.70 (0.23) beats per minute for women and 67.26 (0.28), 65.74 (0.28), 65.56 (0.28), 66.67 (0.28) beats per minute for men.

### Differences between CHD cases and non-cases

Compared to those who remained free of disease, men and women who subsequently developed CHD tended to have higher mean age, blood pressure, glucose and atherogenic lipids at baseline. They also were more likely to be smokers, have hypertension, diabetes, a higher Cornell voltage, minor T wave abnormalities and a lower mean values for the QRS axis in frontal plane. The mean QT interval and the mean T wave axis in frontal plane were not significantly different between cases and non-cases. In contrast to men, women who developed CHD had higher mean values for body mass index, waist to hip ratio, and heart rate at baseline. Spatial T wave axis deviation was only slightly increased among cases compared to non-cases (25.2 degrees versus 23.2 degrees among women, and 24.4 degrees versus 23.9 degrees among men). Mean values for the QT interval, heart rate, or T wave axis in frontal plane were not statistically different.

### Incident CHD events and relative risks of CHD

Overall, the cumulative incidence of CHD was almost uniform across the distribution of the spatial T wave axis deviation, with an increase in the last quartile. The incidence rate was 4.26, 4.18, 4.28 and 5.62 per 1000 person-years across quartiles of spatial T wave axis deviation respectively.

As Table 3 illustrates, the incidence rate ratio was 1.62 (95%CI of 1.15 to 2.27) for women in the upper quartile of the spatial T wave axis deviation compared with women in the bottom quartile. Adjustment for age, height and weight attenuated this ratio to 1.44 (95%CI of 1.02 to 2.04). Among men, the incidence rate ratio comparing the highest with the lower quartile was 1.09 (0.83–1.42), which was unchanged (1.08, 0.83–1.42) after adjustment for age, height and weight.

**Table 3:** Cumulative incidence, incidence density rates and rate ratios for coronary heart disease at 12-years follow-up by spatial T wave axis quartile by gender. The ARIC study

Risk estimate	**Spatial T wave axis deviation quartile among women**			
	
	T axis quartile 1: 0.33–14.75°	T axis quartile 2: 14.75–22.38°	T axis quartile 3: 22.39–30.32°	T axis quartile 4: 30.33–60.88°
Number of event-free participants at baseline	1787	1788	1784	1784
Number of CHD Events	54	57	53	86
Cumulative Incidence [%]	3.02	3.19	2.97	4.82
Person-years	21168	21176	21108	20838
Unadjusted Incidence Rate per 1000 person-years (95% CI)	2.56 (1.87–3.23)	2.70 (1.99–3.39)	2.51 (1.83–3.19)	4.13 (3.25–4.99)
Unadjusted Incidence Rate Ratio (95% CI)	1 (referent)	1.05 (0.72–1.53)	0.98 (0.67–1.44)	1.62 (1.15–2.27)
Adjusted* Incidence Rate Ratio	1 (referent)	1.03 (0.71–1.50)	0.93 (0.64–1.36)	1.44 (1.02–2.04)

Risk estimate	**Spatial T wave axis deviation quartile among men**			
	
	T axis quartile 1 0.19–16.19°	T axis quartile 2 16.19–21.77°	T axis quartile 3 21.77–30.40°	T axis quartile 4 30.19–60.76°

Number of event-free participants at baseline	1281	1276	1279	1277
Number of CHD Events	104	92	96	111
Cumulative Incidence [%]	8.12	7.21	7.51	8.69
Person-years	14699	14605	14603	14410
Unadjusted Incidence Rate per 1000 person-years (95% CI)	7.08 (5.72–8.44)	6.30 (5.01–7.59)	6.57 (5.26–7.89)	7.70 (6.27–9.13)
Unadjusted Incidence Rate Ratio (95% CI)	1 (referent)	0.89 (0.67–1.18)	0.93 (0.70–1.23)	1.09 (0.83–1.42)
Adjusted* Incidence Rate Ratio	1 (referent)	0.90 (0.68–1.20)	0.94 (0.72–1.25)	1.08 (0.83–1.42)

* adjusted for age, height and weight

Cox regression-modeling of the unadjusted association between CHD and spatial T wave axis deviation among women showed that for every 10 degrees increase in the spatial T wave axis deviation, there was a 1.16 (1.04 and 1.28)-fold increase in the risk of CHD (Table 4). When treating spatial T wave axis deviation categorically, borderline and abnormal deviation was associated with a 1.59 (1.20–2.10) and 1.69 (1.04–2.75)-fold higher risk of developing CHD compared to the normal category. When adjusting for age, height, weight, smoking, hypertension, diabetes mellitus, QRS axis and T wave minor abnormalities, this association for women was no longer statistically significant (hazard rate ratio of 1.03 (0.92–1.14). Neither heart rate or the QT interval (regardless of the formula used for heart rate correction), did qualify as confounders, as they were not associated with incident events in this study population.

**Table 4:** Hazard rate ratios (95%CI) of coronary heart disease at 12 years of follow-up for ten degrees increase in the spatial T wave axis deviation and for the comparison of the borderline and/or the abnormal categories with the normal spatial T wave axis category. Data stratified by gender. The ARIC study

Model	Women N = 7143		Men N = 5113	
	
	Continuous T wave axis deviation*	Categorical T wave axis deviation**	Continuos T wave axis deviation*	Categorical T wave axis deviation**
Crude	1.16 (1.05–1.28)	1.59 (1.20–2.10) 1.69 (1.04–2.75)	1.05 (0.96–1.15)	1.11 (0.89–1.40) 1.27 (0.79–2.04)
Adjusted for age (55 yr)	1.15 (1.05–1.27)	1.58 (1.19–2.09) 1.64 (1.01–2.67)	1.05 (0.96–1.15)	1.10 (0.88–1.39) 1.23 (0.76–1.98)
Adjusted for age, height and weight	1.11 (1.00–1.23)	1.46 (1.10–1.94) 1.49 (0.92–2.44)	1.05 (0.96–1.15)	1.10 (0.87–1.39) 1.22 (0.75–1.97)
Adjusted for age, height, weight and QRS axis	1.10 (1.00–1.22)	1.44 (1.09–1.92) 1.47 (0.90–2.40)	1.03 (0.94–1.13)	1.07(0.84–1.35) 1.17(0.72–1.89)
Adjusted for age, height, weight, QRS axis and smoking	1.10 (1.00–1.22)	1.49 (1.12–1.99) 1.42 (0.87–2.32)	1.02 (0.93–1.12)	1.04(0.82–1.32) 1.09 (0.67–1.76)
Adjusted for age, height, weight, QRS axis, smoking, HTN, and DM	1.04 (0.94–1.15)	1.32 (0.99–1.76) 1.06 (0.64–1.77)	0.95 (0.86–1.04)	0.93(0.73–1.18) 0.85(0.52–1.39)

* Spatial T wave axis deviation was treated as a continuous variable and risk estimates are expressed for ten degrees (approximately 1 standard deviation in the distribution) increase in the spatial T wave axis deviation.

** Spatial T wave axis deviation was treated as a categorical variable and risk estimates are expressed for the comparison of the borderline category (30–45 degrees) to the normal category in the first row of the cell and for the comparison of the abnormal category (≥45 degrees) to the normal category (≤30 degrees) in the second row of the cell.

*** HTN and DM indicate hypertension and diabetes mellitus respectively. Hypertension status was defined as systolic blood pressure ≥140 mm Hg or diastolic blood pressure ≥90 mm Hg or current use of antihypertensive medication. Diabetes status was defined as fasting glucose ≥126 mg/dL, nonfasting glucose ≥200 mg/dL, or a physician diagnosis or pharmacological treatment for diabetes.

Among men, there was no significant association between spatial T wave axis deviation and CHD events. This lack of association persisted after adjusting for covariates. T wave axis in the frontal plane was not associated with coronary events either; the crude associations were nil and persisted as such in multivariate analysis in both women and men.

When using the same T wave axis deviation categories as in the CHS and Rotterdam studies, borderline and abnormal deviation was associated with a 1.11 (0.89–1.40) and 1.27 (0.79–2.04)-fold higher risk for developing CHD compared to the normal category. After adjusting for age, height, and weight and as well for smoking, hypertension, diabetes, QRS axis and T wave minor abnormalities, the association remained statistically non-significant. The prevalence of an abnormal spatial T wave axis deviation (larger than 45 degrees) in the ARIC population was extremely low (4.5%) so these risk estimates need to be interpreted with caution.

Minor T wave axis abnormalities were only slightly associated with the risk of developing a coronary heart disease event, in both men and women. The hazard rate ratios for CHD in relation to minor T wave abnormalities were 1.96 (1.37–2.81) for women and 1.31 (0.92–1.88) for men. After adjustment for age, height, weight, smoking, hypertension, diabetes and QRS axis, these estimates became 1.25 (0.86–1.80) for women and 1.02 (0.71–1.47) for men.

The measured (uncorrected for heart rate) QT interval was not associated with coronary events, in either gender group. Incidence rates for coronary events in the first and the last gender-specific QT interval quartiles were 8.04 and 8.00 per 1000 person-years among men and 3.8 and 4.5 per 1000 person-years among women. Adjustment for age and heart rate (either by using the heart rate corrected QT interval by Bazett's formula, QT Index or by including heart rate in the Cox proportional models) did not change this lack of association.

## Discussion

This prospective, population-based, bi-ethnic study of men and women free of coronary heart disease at baseline shows that spatial T wave axis deviation is not associated with incident coronary events during long-term follow-up. The QT interval was not associated, and minor T wave abnormalities were only weakly associated, with the incidence of coronary events after adjustment for various covariates.

Given the lack of association between spatial T wave axis and coronary events in this study, several issues must be considered. Potentially, incomplete ascertainment or misclassification of CHD events could bias the results and obscure an underlying association. The excellent quality control strategies employed by the ARIC study, the highly standardized protocol for data collection, independent case ascertainment through the cohort follow-up, and the epidemiologic surveillance system in place at each of the four ARIC study communities make the possibility of misclassification of coronary heart disease very unlikely.

Inadequate statistical power to detect a meaningful association is not likely, due to the large sample size, long follow-up and relatively large number of events. The minimum effect size that the present study could have missed was computed by dichotomizing spatial T wave axis deviation, then contrasting "borderline" and "abnormal " deviation with the "normal" category (nQuery Advisor, version 4.0 [36]). Given the observed distribution of T wave axis deviation in the present study, a log-rank test of the survival curves at 12 years of follow up would have approximately 79 to 99% power to detect differences between the survival curves corresponding to hazard ratios between 1.11 and 1.22, at a 0.05 two-sided significance level.

Our results do not confirm the findings of the Rotterdam study [23] and the CHS [24] with regard to the spatial T wave axis. The risk of CHD associated with abnormal deviation (more than 45 degrees) versus normal T wave axis deviation (less than 30 degrees) was 2.9 (95% CI 2.0–4.3)) in the Rotterdam study and 1.58 (95%CI 1.25–1.99) in the CHS study. In contrast, findings from the present study are similar to those from the Multiple Risk Factor Intervention Trial (MRFIT) [25], which did not find a significant association between spatial T wave axis deviation at baseline and incident CHD events, although there was a significant relation between change in spatial T axis and long-term mortality from CHD.

The discrepancy between our results and those reported in previous articles has several potential explanations, involving true variation between study populations (differences between study populations in terms of exposure distribution, demographic characteristics, absolute cardiovascular risk level or effect modification by genetic or environmental factors) and different patterns of confounding by other cardiovascular risk factors.

Compared to participants in the present study, those in studies that found a significant association between T wave axis deviation and CHD were older (Rotterdam study and CHS), and more likely to have a history of myocardial infarction, angina pectoris (Rotterdam study) or silent (ECG-diagnosed) myocardial infarction and hypertension at baseline (CHS study). These study populations also had more ECG evidence of ischemia and infarction as reflected by the presence of Q-waves and major ST-T abnormalities (Rotterdam study) or T wave inversion (CHS study) but this was not accounted for these confounders in the analysis. By contrast, the MRFIT study and the present ARIC study excluded these participants, preserving only those with minor T wave abnormalities, and found only weak or non-significant associations with CHD events. Studies reporting stronger associations also had shorter follow-up time. To explore the potential impact of these dissimilarities, we performed several *post hoc *analyses under different scenarios.

### i) without excluding any participants

In comparing individuals without CHD at baseline with prevalent CHD cases at baseline, the spatial T wave axis deviation was significantly different: 26.66 (16.81) and 44.88 (27.29) degrees respectively (p value < .0001). The entire distribution of the spatial T wave axis deviation among those with CHD at baseline was shifted to the right of the distribution of participants without CHD at baseline. This difference persisted statistically significant even after adjustment for age and several standard cardiovascular risk factors (anthropometry, smoking, hypertension, diabetes, heart rate, QRS axis and QT interval) with adjusted mean values of 26.79 and 41.47 degrees respectively. However, among those without CHD at baseline in our study, there were various conditions or markers of clinical interest, some of established predictive value, such as ST segment depression, negative T waves or atrial fibrillation. In the clinical setting, the need for improved prediction and discriminatory ability of a new potentially useful marker is particularly useful in the absence of such markers. After excluding Minnesota codes representing secondary repolarization abnormalities, this difference was markedly attenuated, with mean spatial T wave axis deviation of 23.84 (12.36) for those free of CHD and 24.08 (15.34) degrees for those with CHD. This suggests that spatial T wave axis has no ability to discriminate between CHD cases and CHD-free individuals beyond and above secondary repolarization abnormalities. This might be one of possible explanations for the discrepant results between different cohort studies exploring T wave axis, as different studies have used different exclusion criteria.

As an alternative to the exclusion of Minnesota codes reflecting secondary repolarization abnormalities, we performed a separate analysis this time including QRS duration as a covariate. The relationship of the spatial T wave axis deviation with incident CHD events followed the same pattern as in the restricted dataset, i.e. a slight increase in the incidence rates in the fourth quartile of the T wave axis, for each gender. The crude hazard rate ratios (95%CI) for incident CHD events for a 10 degree increase in the spatial T wave axis were 1.18 (1.13–1.23) for women and 1.14 (1.09–1.19) for men. After adjustment for the same covariates as in the restricted data set and for the QRS duration, the hazard rate ratios (95%CI) were 1.05 (0.99–1.16) and 1.04 (0.98–1.1).

### ii) restricted to fatal events only

Ventricular repolarization abnormalities are closely related to the propensity for arrhythmia. Although none of the published population-based studies on T wave axis deviation attempted to ascertain arrhythmic events, it is plausible that the arrhythmic component of the CHD events was the driving force of the observed associations in some published studies. This suggestion is supported by the fact that in both, Rotterdam and CHS study, the association between T wave axis and coronary events was stronger for fatal than non-fatal events. Similarly, the spatial QRS-T angle was recently reported to be associated with fatal but not with non-fatal cardiac events [37]. To explore this issue we restricted our analysis to fatal coronary events. During an average follow-up time of 11.6 years, 143 fatal events were recorded in our study population, 62 among women and 81 among men, with an overall cumulative incidence of 1.17%. The relationship of fatal events with the spatial T wave axis deviation followed the same pattern as the combined outcome, i.e. a slight increase in the incidence rates for CHD only in the fourth quartile of the spatial T wave axis deviation, for each gender. As expected, in the multivariate models (Table 5), the crude hazard rate ratios for fatal events were slightly larger than for the combined outcome. After adjustment for a limited number of risk factors (due to the small number of events), these modest associations were no longer statistically significant. However, these patterns must be interpreted with caution. While in the Rotterdam and CHS studies, the incidence rate for fatal events was 4.2 (95% CI 3.3–5.1) and 5.8 (95% CI 5.0–6.7) per 1000 person year respectively, this rate was much lower in our study: 1.01 per 1000 person-years (95% CI, 0.84–1.17).

**Table 5:** Hazard rate ratios (95%CI) for fatal and combined, fatal and nonfatal coronary heart disease events at 12 years of follow-up for ten degrees increase in the spatial T wave axis deviation. Data stratified by gender. The ARIC study.

Model	Fatal + non-fatal events		Fatal events	
	
	Women	Men	Women	Men
Crude	1.16 (1.04–1.29	1.05 (0.96–1.15)	1.27(1.04–1.55)	1.24 (1.02–1.50)
Adjusted*	1.06 (0.95–1.17)	0.96 (0.88–1.06)	1.11 (0.91–1.35)	1.09 (0.90–1.33)

*Adjusted for weight smoking HTN and DM

iii) similarly, we repeated the analysis without excluding those with negative T waves corresponding to MC 5.2, restricted to the age group greater than 55 years, restricted to participants with both hypertension and diabetes at baseline, restricted to CHD events occurring after a shorter follow-up (4–6 years). While the incidence rates were larger in absolute value in each T wave axis quartile, in relative terms no association with CHD events was detected.

Experimental models to link the spatial T wave axis deviation with coronary evens are still sparse, but it can be speculated that an abnormal spatial T wave axis may possibly reflect disturbances in the repolarization process caused by subclinical myocardial disease, with or without an increased propensity for arrhythmic events, and thus an increased risk for fatal cardiac events. This is supported by the fact that subclinical disease is likely more prevalent in the older population of the CHS and Rotterdam study (which found an association between T wave axis and incident coronary events) than in the younger populations of the ARIC and MRFIT studies (both reporting a lack of association). Some indicators of subclinical disease such as the left ventricular hypertrophy (LVH) are clearly more prevalent in the Rotterdam than in the ARIC study. The presence of detected or undetected subclinical disease (LVH, patchy myocardial fibrosis) may lead to ventricular electrical instability and a higher proportion of cardiac death related to primary arrhythmic events in a population. Thus, measured or unmeasured, subclinical disease may play a confounding role, and suggests one more explanation for the discrepancy of results between different studies.

The ARIC, CHS and MRFIT study protocols all used the same definition of the spatial T wave axis deviation, while the Rotterdam study used the frontal plane T wave axis. There are no data available to explain the discrepancy of results solely on the basis of different measurement of the T wave axis.

The comparison of spatial T wave axis deviation with its temporal counterpart, the QT interval raises an intriguing question. Incident coronary events were strongly associated with T wave axis in the cohort of older adults of the Rotterdam study, which examined a population of older adults in whom a prolonged QT interval was previously reported to be predictive. In the current work, neither spatial T wave axis deviation nor the QT interval (as measured or heart-rate corrected by different formulae) was associated with incident coronary events.

In our study, a statistical interaction between QT interval and spatial T wave axis deviation was not detected within the range of the QT interval in this population (data not shown). It is important to mention that in the Rotterdam study the mean QT interval (measured with identical methodology as in our study) was considerably larger than in the present study. From electrophysiological point of view, an abnormal ventricular repolarization can alter the QT interval, the T wave axis or both. It is therefore possible that a given magnitude of an altered spatial T wave axis in the presence of a QT interval within "the normal" ranges, may not be sufficient to elicit any causal association with incident coronary events.

Our study found an inverse association between spatial T wave axis deviation and heart rate. Heart rate influences have been reported on the frontal plane T wave angle [18] and on the spatial ST-T vector [38]. The physiologic explanation of the dependency of an angular measure on heart rate is not clear. It is plausible that this phenomenon is similar to some degree with that observed for another index of altered repolarization, namely T wave alternans, characterized by the change in amplitude, morphology and axis of the spatial T wave and associated with an increased risk of sudden cardiac death. Even if the mechanism of heart rate dependency of the spatial T wave axis remains elusive, it raises questions about the need for "rate correction" of this measurement.

Our study confirms the results of other studies [18,19] in finding a strong association between spatial T wave axis and hypertension status. The explanation is likely related to the presence of an increased left ventricular wall in the context of the left ventricular hypertrophy. It is also possible that increased spatial Twave axis deviation is related to other processes present in the hypertensive myocardium, such as microfocal areas of fibrous tissue and/or increased alteration of ionic channels. These findings suggest that spatial T wave axis deviation may serve as an auxiliary early marker of repolarization abnormalities in hypertensive individuals. Due to the low prevalence of left ventricular hypertrophy in our study population, we were not able to further explore this issue.

## Strengths and limitations

The present study has several strengths. The highly standardized data collection procedure and ECG protocol increase the internal validity of the findings. The ECG recording was performed by trained technicians using a standardized protocol for lead placement. The events ascertainment in the ARIC study is ensured by a highly standardized quality assurance protocol. The inclusion of apparently healthy participants in a population-based study, of African American and white men and women, the large sample size and the geographic distribution of the study population enhance generalizability.

This study also has some limitations. The narrow age range precluded an extensive investigation of the role of age. The study also was limited by the lack of accurate measurements for characterizing the elliptical shape of the thoracic cavity. Constitutional and anthropometric variables such as the chest transverse diameter or chest shape have been associated with a shift of the T wave axis [39] or changes in average T wave potential amplitudes. A slender body with a limited spatial sampling of the thorax directly overlaying the heart can cause lower amplitudes in women than in men [40]. While our study attempted to account for this fact by adjusting for height and weight, a more accurate measurement of thoracic cavity, shape or body surface area would probably result in a better ascertainment of spatial T axis. Nonetheless, previous studies reporting positive findings did not account for the shape of thoracic cavity either, and it is unlikely that such measurement would have influenced the findings of the present study.

Ventricular replarization is a complex process, which cannot be fully captured by only a limited number of indexes such as QT interval and spatial T wave axis, exploring either the temporal aspects or the general direction of this process. Other indexes or more complex descriptors of the T wave loop have been described to be accurate markers of an altered repolarization [41]. The spatial QRS-T angle has recently regained attention and several studies have attested its usefulness in post infarction [12] and hypertensive patients [19]. The spatial QRS-T angle was also the strongest predictor of fatal cardiac events, even after controlling for the frontal plane T axis, in the large population-based cohort of men and women aged 55 years or older of the Rotterdam study [37]. Unfortunately, this index is not currently available in our database.

## Conclusions

In conclusion, this prospective, population-based, bi-ethnic study of men and women free of coronary heart disease at baseline shows that spatial T wave axis deviation is not associated with incident coronary events during long-term follow up. It is doubtful that T wave axis deviation would add benefit in the prediction of CHD events above and beyond the current traditional risk factors. The QT interval was not associated with incident coronary events, and minor T wave abnormalities were only weakly associated among women.

## Competing interests

The author(s) declare that they have no competing interests.

## Authors' contributions

GV performed the overall study design, statistical analysis and manuscript preparation.

PR designed the data collection protocol, participated in the interpretation of the ECG data, provided comments on the manuscript.

RP participated in the quality control of the ECG data, participated in the interpretation of the findings and with comments on the manuscript.

EAW participated in the interpretation of the findings and with comments on the manuscript, provided assistance in the manuscript preparation.

LEC provided oversight of the statistical analysis, verified the appropriateness of statistical models, provided assistance in the manuscript preparation.

ARF participated in the interpretation of the findings and with comments on the manuscript, provided assistance in the manuscript preparation.

WDR collaborated in the study design, participated with comments on the manuscript.

ZMZ managed the automated coding of ECGs and the quality assurance.

RC participated with comments on the manuscript.

GH collaborated in the design of the study, provided comments on the manuscript.

## Pre-publication history

The pre-publication history for this paper can be accessed here:


